# Critical comparison of methods for fault diagnosis in metabolomics data

**DOI:** 10.1038/s41598-018-37494-7

**Published:** 2019-02-04

**Authors:** M. Koeman, J. Engel, J. Jansen, L. Buydens

**Affiliations:** 1 Radboud University, Institute for Molecules and Materials (IMM) Heyendaalseweg 135, 6525 AJ Nijmegen, The Netherlands; 2 Biometris, Wageningen UR, Droevendaalsesteeg 1, 6708 PB Wageningen, The Netherlands

## Abstract

Platforms like metabolomics provide an unprecedented view on the chemical versatility in biomedical samples. Many diseases reflect themselves as perturbations in specific metabolite combinations. Multivariate analyses are essential to detect such combinations and associate them to specific diseases. For this, usually targeted discriminations of samples associated to a specific disease from non-diseased control samples are used. Such targeted data interpretation may not respect the heterogeneity of metabolic responses, both between diseases and within diseases. Here we show that multivariate methods that find any set of perturbed metabolites in a single patient, may be employed in combination with data collected with a single metabolomics technology to simultaneously investigate a large array of diseases. Several such untargeted data analysis approaches have been already proposed in other fields to find both expected and unexpected perturbations, e.g. in Statistical Process Control. We have critically compared several of these approaches for their sensitivity and their correct identification of the specifically perturbed metabolites. Also a new approach is introduced for this purpose. The newly introduced Sparse Mean approach, which we find here as most sensitive and best able to identify the specifically perturbed metabolites, turns metabolomics into an untargeted diagnostic platform. Aside from metabolomics, the proposed approach may greatly benefit fault diagnosis with untargeted analyses in many other fields, such as Industrial Process Control, food Adulteration Detection, and Intrusion Detection.

## Introduction

MSPC (Multivariate Statistical Process Control) type approaches^1,2^ use a multivariate model to describe data from observations that have been defined as ‘normal’ or control. They use this model to identify in new observations those that do not fit this model and are thus ‘abnormal’. This approach is also known as one-class modeling. Applications include Industrial Process Control^3^, Adulteration Detection^4^, Intrusion Detection^5^ and Health Monitoring^6,7^. These are all situations where the training “group” of abnormal samples contains no, or relatively few, observations compared to the large number of control observations, a scenario where two or multi-class classification performs poorly. One-class modeling also allows for detection of *heterogeneous* abnormalities where a phenomenon may cause different reactions in different observations. This is especially beneficial in the case of the aforementioned Health Monitoring which applies MSPC to metabolomics data to identify molecular perturbations due to possibly heterogeneous diseases. These heterogeneous diseases, such as asthma, are typically hard to classify in the traditional manner^8^. While the observations in this work are applicable in general, we will focus on the analysis of metabolomics in the context of Health Monitoring.

After the first step of detecting abnormal samples, or ‘identifying’ presence of an abnormality, the second step is to ‘diagnose’ why that sample is abnormal. This step aims at providing insight into the potential root cause of the abnormality and is often referred to as fault diagnosis. To make diagnosis of a disease (a fault) as reliable as possible, the set of variables on which the ‘abnormal’ observation deviates from the control observations should be identified as correctly as possible. Ideally, no false positive variables should be identified, to avoid false alarms, but also the full complement of deviating variables should be identified to warrant accurate root cause identification. For example, the fault diagnosis step in Health Monitoring can give valuable clues to which disease (if any) the evaluated individual might be suffering from. The strength of untargeted ‘omics’ methods, like metabolomics, is that they provide a broad-spectrum view on the chemical composition of a biofluid. Therefore, fault identification and diagnosis combined with such analytical technologies may provide a diagnosis in which a large array of different diseases—both known and unknown—could be simultaneously investigated^7^. As much work has been done towards the detection step^9^ and fewer towards the diagnosis, we restrict ourselves to the latter.

In this work we will investigate and adapt several recently proposed fault diagnosis methods and compare them to more conventional alternatives to asses which provide the most accurate diagnosis. Classical MSPC^1^ is performed by applying Principal Component Analysis (PCA) to data of a set of control samples. This separates the variation between these control samples into a subspace spanned by the selected Principal Components (PC) and a ‘residual’ subspace. New, potentially abnormal, samples can then be tested by projection onto this PCA model and either subspaces can then be tested for compliance with those of the control samples with a suitable statistical test. Subsequently, samples that deviate significantly from the known control samples (i.e. outliers) are further investigated using fault diagnosis approaches, that quantify and decompose the deviation observed in either of the two subspaces into variable-specific contributions. The contribution-values are typically visualized in a so-called contribution plot^10^, where large contributions indicate ‘abnormal’ variables.

The use of classical MSPC is widespread, but has a number of limitations. First, geometrically, an abnormality can be understood as having a specific direction in multivariate space, possibly partly in both the PC-subspace and in the residual subspace, which makes the separation into two subspaces artificial. Specifically in metabolomics, disease-associated processes may not manifest completely in the healthy space, as they are processes that are by definition not healthy. It may be reasoned that they may not be completely described by the residual subspace either as the disease will also affect the healthy metabolism. A second issue is determining the optimal number of principal components as this controls the split in the two subspaces. There may be no suitable objective criterion available to optimize this critical parameter. Finally, there is the ‘smearing effect’^11,12^, which may diagnose non-abnormal variables as false positives, due to the geometry of the aforementioned partition of variability in the dimension reduction, which may greatly limit the interpretability of the contribution plots in terms an interpretable root cause.

Circumventing the dimension reduction may intuitively avoid all these concerns. We investigate three (recently proposed) approaches: ‘serial univariate analysis’ using *Z*-scores, Whitening, followed by a *Z*-score analysis and a forward variable selection method^13^ that imposes sparsity on the difference with the control group. All of these methods assume that a disease causes a shift in some variables with respect to the mean of the control population, which is also the case we restrict ourselves to. Were required we have adapted these methods to the high-dimensional case using shrinkage estimation of the covariance matrices involved, thus avoiding the need for dimension reduction suitable for high-dimensional data, without dimension reduction.

We compare these methods to conventional PCA-based MSPC in several simulation studies. We only consider the diagnosis step here and assume the samples have already been identified as being abnormal. For more information MSPC we refer the reader to^9,14–16^. We first impose faults directly on simulated multivariate data of the controls to study the identification of the variables that were perturbed as a function of the correlation structure between the variables. Secondly, we created more realistic metabolic perturbations by perturbing enzyme activities in a metabolic network reconstruction of glycolysis in yeast^17^. In this case, Metabolic Control Analysis was used to define a gold standard of perturbed metabolites to which the results of the fault diagnosis methods were compared^18,19^. Finally, it is demonstrated how fault diagnosis in combination with ^1^H-NMR untargeted metabolomics can be used for diagnosis of an inborn error of metabolism.

## Methods

Throughout the rest of this work we use three assumptions. First, we assume multivariate normal distributions of the both the control and abnormal data (possibly after a suitable transformation^20^ such as a log transform or generalized log transform) with mean **μ** and covariance ${\boldsymbol{\Sigma }}$. Secondly, we assume that the abnormality manifests itself as a deviation from the mean of the normal group i.e. the abnormal data has the same covariance ${\boldsymbol{\Sigma }}$ but a different mean, **μ** − **μ**_a_. Finally, we assume that the abnormality occurs only in a limited number of variables, i.e. that abnormalities, **μ** − **μ**_a_, are sparse in multivariate space. This final assumption makes sense in a metabolics application as typically diseases do not affect the *entire* metabolome.

### Mahalanobis Distance

All methods we will be discussing here are in one way or another based on the squared Mahalanobis distance (MD) between a sample (**x**) and the control group, see eq. (1)

1 $${D}_{m}^{2}({\bf{x}})=({\bf{x}}-{\boldsymbol{\mu }}){{\boldsymbol{\Sigma }}}^{-1}{({\bf{x}}-{\boldsymbol{\mu }})}^{{\rm{T}}}.$$

Where **x** is the 1 × *m* sample vector to be tested, **μ** the 1 × *m* vector with the control means and ${\boldsymbol{\Sigma }}$ is the *m* × *m* population covariance matrix where *m* is the number of variables. More dissimilar samples, i.e. larger values for ${D}_{m}^{2}$, are more abnormal. To calculate the MD we replace the unknown ${\boldsymbol{\Sigma }}$ and **μ** in (1), with their sample estimates $\overline{{\bar{{\bf{x}}}}_{c}}$ and **S**. Subsequently, a cutoff value for abnormality can be determined from an *F*-distribution^21^. There are two concerns with using the Mahalanobis in this manner. First of all, **S** is often rank deficient for high-dimensional data and cannot be inverted. This requires a more advanced estimator of ${\boldsymbol{\Sigma }}$. Secondly, the MD cannot readily be used for fault diagnosis as it is a single number for each test sample; no knowledge regarding in which variables the abnormality is encountered is obtained directly. We will discuss multiple ways to address these concerns.

### Approach 1: Conventional Multivariate Statistical Process Control

Perhaps the most straightforward approach to circumvent the rank deficiency of **S** is to do a dimension reduction. This leads to the classical Multivariate Statistical Process Control (MSPC)^2^.

We derive MSPC from the Mahalanobis distance eq. (1) where we replace ${{\boldsymbol{\Sigma }}}^{-1}$ by its eigenvalue decomposition (PCA) *P***Λ**^−1^**P**^T^ to obtain

2 $${D}_{m}^{2}({\bf{x}})=({\bf{x}}-{\boldsymbol{\mu }}){\bf{P}}{{\boldsymbol{\Lambda }}}^{-1}{{\bf{P}}}^{{\rm{T}}}{({\bf{x}}-{\boldsymbol{\mu }})}^{{\rm{T}}},$$

where **P** is a (*m* × *m*) loading matrix and **Λ** a (*m* × *m*) diagonal matrix with the eigenvalues. We proceed to approximate eq. (2) as a sum of the selected PC’s and the residual PC’s.

3 $${D}_{m}^{2}({\bf{x}})\cong ({\bf{x}}-{\boldsymbol{\mu }})\bar{{\bf{P}}}{\bar{{\boldsymbol{\Lambda }}}}^{-1}{\bar{{\bf{P}}}}^{{\rm{T}}}\,{({\bf{x}}-{\boldsymbol{\mu }})}^{{\rm{T}}}+({\bf{x}}-{\boldsymbol{\mu }})\tilde{{\bf{P}}}{\tilde{{\bf{P}}}}^{{\rm{T}}}{({\bf{x}}-{\boldsymbol{\mu }})}^{{\rm{T}}},$$

where $\bar{{\bf{P}}}$ is a (*m* × *K*) loading matrix consisting of the selected PC’s, $\bar{{\boldsymbol{\Lambda }}}$ a (*K* × *K*) diagonal matrix with their corresponding eigenvalues and $\tilde{{\bf{P}}}$ the (*m* × (*m* − *K*)) loading matrix for the residual PC’s where *K* is the number of selected components. Here we have made the assumption that the eigenvalues for the residual loadings are equal to 1. In other words, we approximate the Mahalanobis distance as the sum of the Mahalanobis distance of the scores in the PC-subspace and the Euclidian distance of the residuals. Finally, we split this sum up into a *T*^2^ part and a *Q* part.

4 $${D}_{{T}^{2}}^{2}({\bf{x}})=({\bf{x}}-{\boldsymbol{\mu }})\bar{{\bf{P}}}{\bar{{\boldsymbol{\Lambda }}}}^{-1}{{\bf{P}}}^{{\bf{T}}}\,{({\bf{x}}-{\boldsymbol{\mu }})}^{{\rm{T}}}$$

and

5 $${D}_{Q}^{2}({\bf{x}})=({\bf{x}}-{\boldsymbol{\mu }})\tilde{{\bf{P}}}{\tilde{{\bf{P}}}}^{{\rm{T}}}{({\bf{x}}-{\boldsymbol{\mu }})}^{{\rm{T}}}.$$

Equations (4) and (5) can both be used to detect abnormal samples. The fault diagnosis step can be done with the use of so-called contribution plots. These decompose either the *Q* or *T*^2^ statistic into variable-wise contributions in various ways. Here we consider two ways; the Complete Decomposition (CDC) and Partial Decomposition (PDC) for the *i*-th variable are given below:

6 $${\rm{CDC}}(i)={({{\boldsymbol{\xi }}}_{{\rm{i}}}{{\bf{M}}}^{\frac{1}{2}}{{\bf{x}}}^{{\rm{T}}})}^{2}$$

and

7 $${\rm{PDC}}(i)={\bf{x}}{\bf{M}}{{\boldsymbol{\xi }}}_{{\rm{i}}}{{\boldsymbol{\xi }}}_{{\rm{i}}}^{{\rm{T}}}{{\bf{x}}}^{{\rm{T}}}.$$

Where **ξ**_i_ is the *i*-th column of the identity matrix and **M** is either $\bar{{\bf{P}}}{\bar{{\boldsymbol{\Lambda }}}}^{-1}{\bar{{\bf{P}}}}^{{\bf{T}}}$ for Hoteling’s *T*^2^ or ${\bf{I}}-\bar{{\bf{P}}}{\bar{{\bf{P}}}}^{{\rm{T}}}$ for *Q*. Again, the higher the value the more abnormal the variable is. Other types of contribution plots have also been proposed in the literature such as the reconstruction based or angle based among others, but we consider only the PDC and CDC here as they the most commonly applied in practice.

### Regularization as a generic approach to avoid rank deficiency

A second way to use the Mahalanobis distance in high-dimensional datasets is to apply a regularized estimator of the covariance matrix as in eq. (8)

8 $${{\bf{S}}}_{{\rm{reg}}}=(1-\lambda ){\bf{S}}+\lambda \,{\bf{d}}{\bf{i}}{\bf{a}}{\bf{g}}({\bf{S}}).$$

Where *λ* is the constant that determines how much regularization is applied and **diag**(**S**) is a diagonal matrix with the control group variances. Here, we are shrinking the covariance matrix toward the uncorrelated situation according to *λ*. The right value of *λ* allows one to invert **S**_reg_ even in situations with more variables than control samples, and, generally, **S**_reg_ is a more accurate estimate of ${\boldsymbol{\Sigma }}$. The optimal value for *λ* can be determined analytically which we have done according to the approach by Schäfer^22^. For the remainder of this section we assume that ${\boldsymbol{\Sigma }}$ can either be estimated sufficiently well by its sample estimate **S** or the regularized version, **S**_reg_, has been used instead.

### Approach 2: Serial univariate diagnosis with *Z*-scores

One approach to circumvent direct estimation of **S** from ${\boldsymbol{\Sigma }}$ is assume that ${\boldsymbol{\Sigma }}={\bf{D}}$ in eq. (1), where **D** is a *m* × *m* diagonal matrix with the control variances on the diagonal for each variable. This chooses *λ* from the previous paragraph (eq. 9) equal to 1. Note that a diagonal covariance structure implies that all variables are uncorrelated. With this assumption we can simplify (see Supplementary Materials 1) eq. (1) to

9 $${D}_{m}^{2}=\sum _{i=1}^{M}\,{Z}_{i}^{2}\,{\rm{with}}$$

10 $${Z}_{i}=|\frac{{x}_{i}-{\mu }_{i}}{{\sigma }_{i}}|,$$

where *Z*_*i*_ is the *Z*-score for variable *i*, and *σ*_*i*_ is the standard deviation of variable *i*.

By assuming ${\boldsymbol{\Sigma }}={\bf{D}}$ we have simplified eq. (1) to the Euclidean distance. *Z*_*i*_ is now a measure of variable abnormality and corresponds to the *Z*-score. The values of *Z*_*i*_ may now be ranked to identify the variables that deviate most from the control group (largest *Z*-scores). Note that the *Z*-scores from eq. (10) are up to a constant equal to the two sample *t*-test statistic for testing the difference between the means of the controls and patient population and can therefore be compared to a students *t*-distribution (under the null hypothesis of no difference) to assign statistical significance to them. The number of false positives identifications can be controlled by application of a multiple testing correction procedure such as the Benjamini-Hochberg False Discovery Rate^23^. An advantage of the assumption ${\boldsymbol{\Sigma }}={\bf{D}}$ is the ease of computation of eqs (9 and 10) and the fact that strict FDR-control for identification of the abnormal variables is possible. At the same time, however, the method may suffer from poor power for identification of abnormal samples and subsequent fault diagnosis of abnormal variables when the diagonality assumption is not met, i.e. for general covariance structures ${\boldsymbol{\Sigma }}$.

### Approach 3: Whitening

As a (to the best of our knowledge) new approach, we propose to use whitened Z-scores for fault diagnosis. In Whitening, we consider only part of eq. (1) for identification of abnormal variables:

11 $${\bf{w}}=({\bf{x}}-{\boldsymbol{\mu }}){{\boldsymbol{\Sigma }}}^{-\frac{1}{2}}.$$

Where **w** is the 1 × *m* vector that contains the measure of abnormality for each variable. Here we have taken only a part of eq. (1) by writing ${{\boldsymbol{\Sigma }}}^{-1}$ as ${{\boldsymbol{\Sigma }}}^{-\frac{1}{2}}{{\boldsymbol{\Sigma }}}^{-\frac{1}{2}}$. Note that ${{\bf{w}}}^{2}={\bf{w}}{{\bf{w}}}^{{\rm{T}}}={D}_{m}^{2}$ from eq. (1). The values in **w** can be ranked based on their absolute value to identify the most abnormal variables. Here, we again used the shrinkage approach described earlier to improve our estimation of ${\boldsymbol{\Sigma }}$.

Note that this approach essentially combines ‘Whitening’ the data, decorrelation of the data by multiplication of (**x** − **μ**) by ${{\boldsymbol{\Sigma }}}^{-\frac{1}{2}}$, with the *Z*-scores approach. In other words, to ensure that the assumption of uncorrelated variables in method 1 is met, the data is first decorrelated. As discussed in Kessy *et al*.^24^, there exists no unique way to decorrelate a data matrix. The approach used here ensures that the variables in the Whitened data are maximally correlated to the variables in the original data^24^. This accomplished by a rotation of the data by multiplying the data with ${{\boldsymbol{\Sigma }}}^{-\frac{1}{2}}$. Thus, if a variable in the Whitened data space is marked as abnormal, most likely the same variable was abnormal in the original space. As the variable abnormality is calculated analogous to the *Z*-score, a list of (multiple testing corrected) significant variables may also be determined for this whitening approach.

### Approach 4: Sparsity on the deviation of the mean

The final method of using the Mahalanobis distance for diagnosis is by directly combining estimation of the distance with variable selection. This is achieved by placing an L_0_-norm constraint on the vector (**μ**_a_) of the difference between the sample and the mean of the controls, resulting in eqs (12) and (13)

12 $${D}_{m}^{2}({\bf{x}})={{\boldsymbol{\mu }}}_{{\rm{a}}}{{\boldsymbol{\Sigma }}}^{-1}{{\boldsymbol{\mu }}}_{{\rm{a}}}^{{\rm{T}}}\,$$

with

13 $${{\boldsymbol{\mu }}}_{{\rm{a}}}=\mathop{{\rm{\arg }}\,{\rm{\min }}}\limits_{{{\boldsymbol{\mu }}}_{{\bf{a}}}}\,(({\bf{x}}-{\boldsymbol{\mu }})-{{\boldsymbol{\mu }}}_{{\rm{a}}})\,{{\boldsymbol{\Sigma }}}^{-1}{(({\bf{x}}-{\boldsymbol{\mu }})-{{\boldsymbol{\mu }}}_{{\rm{a}}})}^{{\rm{T}}}$$

$${\rm{s}}.{\rm{t}}.\,\sum _{i=1}^{N}\,I(|{\mu }_{i}|\ne 0)\le \tau ,$$

where **μ**_a_ is a 1 × *m* sparse vector containing the variable abnormalities i.e. all non-zero values are abnormal variables, and *τ* is a constant that determines how many non-zero variables are selected. Eq. (13) attempts find the vector of variable abnormalities which can then be used in eq. (12) to calculate the Mahalanobis distance. Note that in the case that all variables are selected (*τ* = *m*), eq. (12) simplifies to eq. (1).

The sparsity constraint makes sense here since we assumed that the abnormality is sparse with respect to the difference between the means of the control and patient populations. Equtation (13) can be solved in multiple ways^13,25^, we have opted to use the Forward Selection approach as proposed by Wang *et al*.^13^. This approach rewrites eq. (13) to a regression problem which can then be solved by Forward Selection. The key difference between this approach and approach 1 and 2 is that the ranking step is not required. Instead of determining the abnormality index per variable, we determine which variables are abnormal given that there are *τ* abnormal variables which may lead to a different ranking (by order of selection) of the variables.

Note that, recently, other penalizations of the Mahalanobis distance have been proposed as well. For example, Capizzi *et al*.^25^ use an expression similar to eq. (13), but replace the L_0_-norm constraint is by an L_1_-norm constraint. This essentially reduces the problem to a LASSO-problem, which can be solved using standard routines such as that by Tibshirani^26^. Engel *et al*., propose a slightly difference penalization where it is assumed that ${{\boldsymbol{\Sigma }}}^{-1}({\bf{x}}-{\boldsymbol{\mu }})$ rather than **x** − **μ** is sparse^27^. This seems like an intuitive approach since the direction along which abnormal samples differ from the controls in multivariate space is penalized directly this way. In the present study we only consider simulations where **x** − **μ** rather than ${{\boldsymbol{\Sigma }}}^{-1}({\bf{x}}-{\boldsymbol{\mu }})$ is sparse. Therefore, it is no surprise that the method of Engel *et al*. performed quite poorly (results not shown). As will be discussed more thoroughly in the discussion section, an interesting avenue for future research is to determine which penalization is most sensible for a specific application (e.g. Health Monitoring).

The high-dimensional extension of both Whitening, and what we call Sparse Mean by regularization using eq. (8) has not been done before to our knowledge. As these methods do not use a dimension reduction, they, by definition, do not suffer from the smearing effect, which may lead to a more accurate analysis.

### Simulations

We compare these approaches 1–4 in a simulation study of multivariate normal data with several perturbation scenarios. Next, data generated from metabolic network reconstruction of the glycolysis of Saccharomyces Cerevisiae is used to assess how well, based on steady state concentrations, the methods are able to identify abnormal metabolites due to perturbation in the activity of a specific enzyme. Finally, ^1^H-NMR spectra of urine are analyzed by Sparse Mean to diagnose an inborn error of metabolism.

### Simulation of multivariate data

We have performed a simulation study to assess the methods described in the method section. We are going to assume that the test samples have already been identified as being abnormal and are only concerning ourselves with finding out why these samples are abnormal. The methods we have used are the *Z*-score, the Sparse Mean, Whitening and the PCA-based methods, both the *Q*- and *T*^2^-statistic with the both the PDC and CDC contribution plots.

We have independently drawn control samples from ${\mathbb{N}}(0,{\bf{R}})$ and test samples from ${\mathbb{N}}({{\boldsymbol{\mu }}}_{a},{\bf{R}})$ where **μ**_*a*_ is a sparse vector with *l* non-zero elements of constant magnitude that gives a ‘shift’ to the test samples when compared to the control samples and **R** a correlation matrix. We varied the magnitude of **μ**_*a*_, **R** and to which variables the abnormality was introduced but kept the number of variables fixed at 100. In the results we are showing we have always used 100 control samples and 1000 test samples and 2 abnormal variables. We are using different correlation structures for our simulations, one of them is a simple block correlation with 10 blocks each consisting of 10 variables with a correlation of 0.8 to each other. The second structure is a slightly more complex block correlation with varying amounts of correlation and block sizes. The last structure was based on the correlation matrix of an LC-MS metabolomics data set from the HUSERMET project, where we have used the pairwise correlations between the 100 variables with the highest variance. The number of principal components was determined using the NUMFACT method^28^. We have visualized these correlation structures next to the results of these correlation structures in the result section.

### Performance criterion

To estimate how well the methods are performing compared to each other we are using the percentage correct diagnosis which is defined for a single sample by:

14 $$q\equiv \frac{100}{l}\,\sum _{i=1}^{l}\,\sum _{j=1}^{l}\,\,\delta ({a}_{i},{b}_{j})$$

where *l* is the number of abnormal variables, *a*_*i*_ the *i*-th true abnormal variable, *b*_*j*_ the *j*-th determined abnormal variable and *δ* the Kronecker delta function. This compares the set of all true abnormal variables with the set determined abnormal variables of the same size and calculates the percentage of overlap. The mean of *q* over all test samples gives the performance. Confidence intervals were constructed by repetition of the simulations but we found that most variation was caused by the different control and test data. This caused some confidence intervals to overlap even though one of the respective methods has a higher performance than the other in all repetitions. These confidence intervals were therefore highly misleading and cluttered the figures on top of that. For this reason, we opted to occasionally report how often the methods outperformed each other instead to give the reader insight in their relative performances.

### Data from metabolic network reconstruction

To provide a more realistic test case we simulated a metabolic network using the simulation software COPASI^18^. We used the network in which describes the glycolysis of Saccharomyces Cerevisiae. To simulate variability in the training samples we varied the initial concentrations of the metabolites by sampling these concentrations from a normal distribution with the mean being the value given by as default in^17^ and a standard deviation of 10% of this mean. The concentrations of each sample were simulated until steady state was achieved and subsequently stored in a data matrix in which each variable corresponded to a specific metabolite.

The test samples have been simulated with the same procedure as the training samples, but the reaction rate catalyzed by pyruvate decarboxylase has been gradually decreased by decreasing the parameter *Vmax_12* from 857.8 to 832.1 in 7 equidistant steps. This reaction converts pyruvate into acetaldehyde and carbon dioxide. Metabolic Control Analysis was performed in COPASI and it was found that decreasing this reaction speed increases the concentration of pyruvate in steady state. Acetaldehyde, the reaction product, was not influenced by this change. As pyruvate was the only concentration that significantly changed according to this analysis we use it as the ground truth for our abnormal variable.

The steady state concentrations were autoscaled and Gaussian noise (*σ* = 0.1) was added to represent a measurement error. The number of components in the training data was determined using the NUMFACT^28^ algorithm and was found to be 4.

### NMR data

To demonstrate the Sparse Mean approach on real data, we used ^1^H-NMR data on urine of children^7^. Outliers were removed after which 118 healthy samples remained. The 246 bins were autoscaled and the regularization constant *λ* was estimated at 0.077. The Sparse Mean approach was applied using a sparsity of 4 variables. For more details regarding the collection of data refer to Engel *et al*.^7^.

## Results

### Block correlation

**Figure 1:**
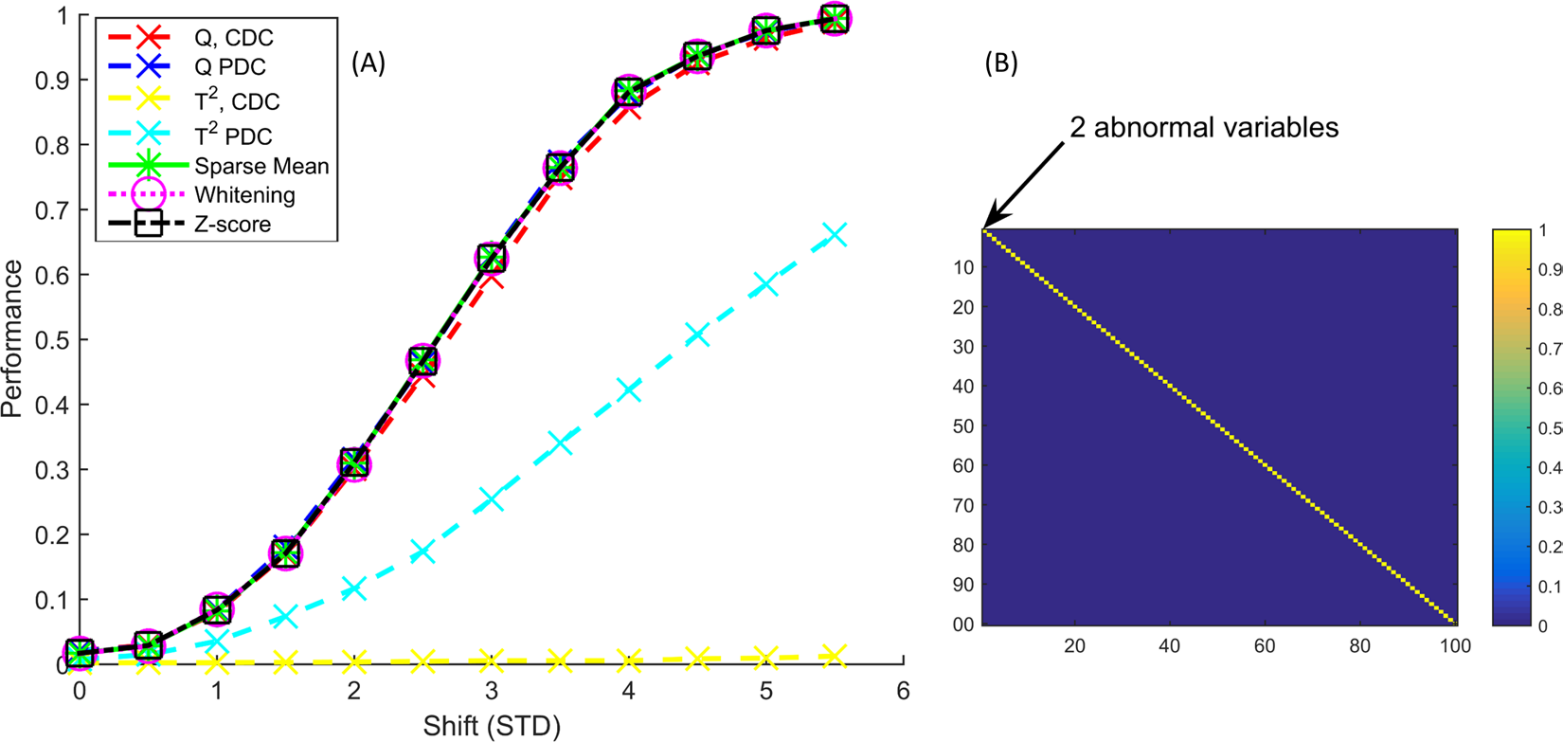
Results (**A**) for identity correlation matrix (**B**) where the first two variables have been perturbed. On the x-axis is the magnitude of the shift in standard deviations and the y-axis is the percent correct diagnosis as defined by eq. (15). All methods recognize the abnormal variables comparably well with exception of partial and complete decomposition of the Hoteling *T*^2^.

We designed our simulations starting with a very simple correlation structure, which was then made increasingly more complex. The simplest structure possible is the identity correlation matrix, where we made 2 variables abnormal (Fig. 1). On the x-axis in Fig. 1 is the magnitude of the shift, **μ**_a_, and the y-axis is *q*, the percent correct diagnosis as defined by eq. (14). All methods identify the abnormal variables comparably well, except for the much poorer performance of Hotelling *T*^2^ which performs poorly when combined with Partial Decomposition (PDC) and even worse for Complete decomposition (CDC). This may be explained by the orthogonality of the introduced abnormality to the PCA model plane: indeed, the Q-residual is much more sensitive to the perturbation. Other correlation structures we subsequently studied show similar lack in performance, we will therefore omit subsequent results for the *T*^2^. As expected, all other methods perform comparably. Specifically, the *Z*-score does not outperform the others, despite meeting the relatively strong assumptions required for this method in this specific simulation setting. This could be a consequence of the fact that a shrinkage estimator was used for the Whitening and Sparse Mean approaches and *λ* was chosen close to 1, meaning that the estimate of the covariance matrix was nearly diagonal. The same simulation with 30 control samples with 2000 variables in 10 blocks is shown in the Supplementary Materials.

**Figure 2:**
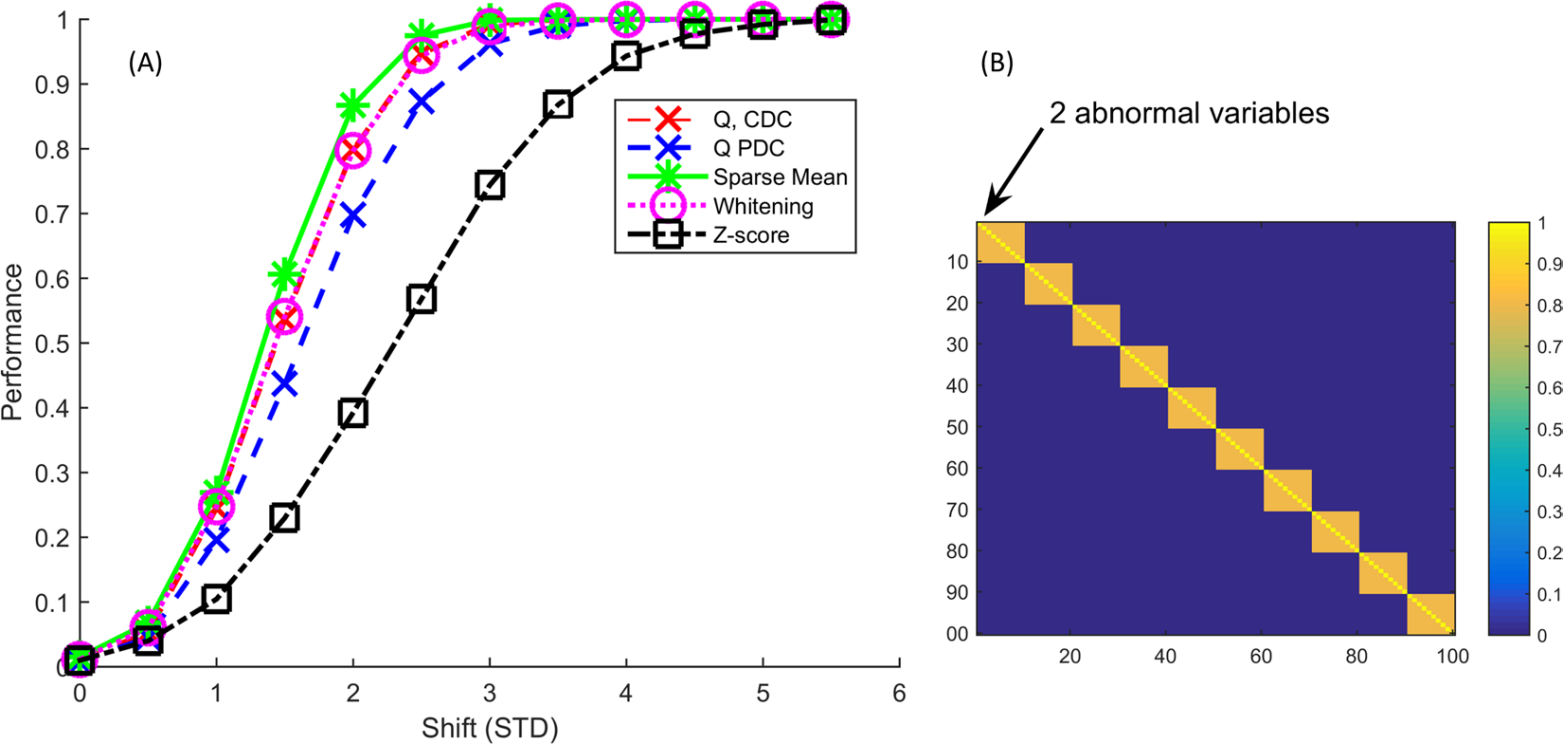
Results (**A**) for the block correlation matrix (**B**) with 10 blocks consisting of 10 variables each with a within block correlation of 0.8. The first two variables have again been perturbed here. This plot shows that the multivariate methods are better able to recognize the true abnormal variables and the Sparse Mean finds the abnormal variables maximally 7% more often. When repeating this multiple times, the Sparse Mean outperforms the *Q* and whitening every time between a shift of 1.5 and 2.5.

The correlation structure consisting of blocks of variables, where the within block correlation is 0.8 is shown in Fig. 2. We first perturbed two variables within the same block. Here, all methods outperform *Z*-score, which is in line with the multicollinear structure in which the perturbation now exists. For this reason, we would advise against using this approach on real data, or any other univariate method for that matter as the strong assumption that all variables are uncorrelated is simply not met. The Sparse Mean method outperforms all methods, although the relative gain in percentage correct diagnosis is limited. Notably, the CDC does better than PDC, even though PDC should suffer less from variable smearing^12^. Whitening performs on par with CDC for this correlation structure.

### More complex block correlation

**Figure 3:**
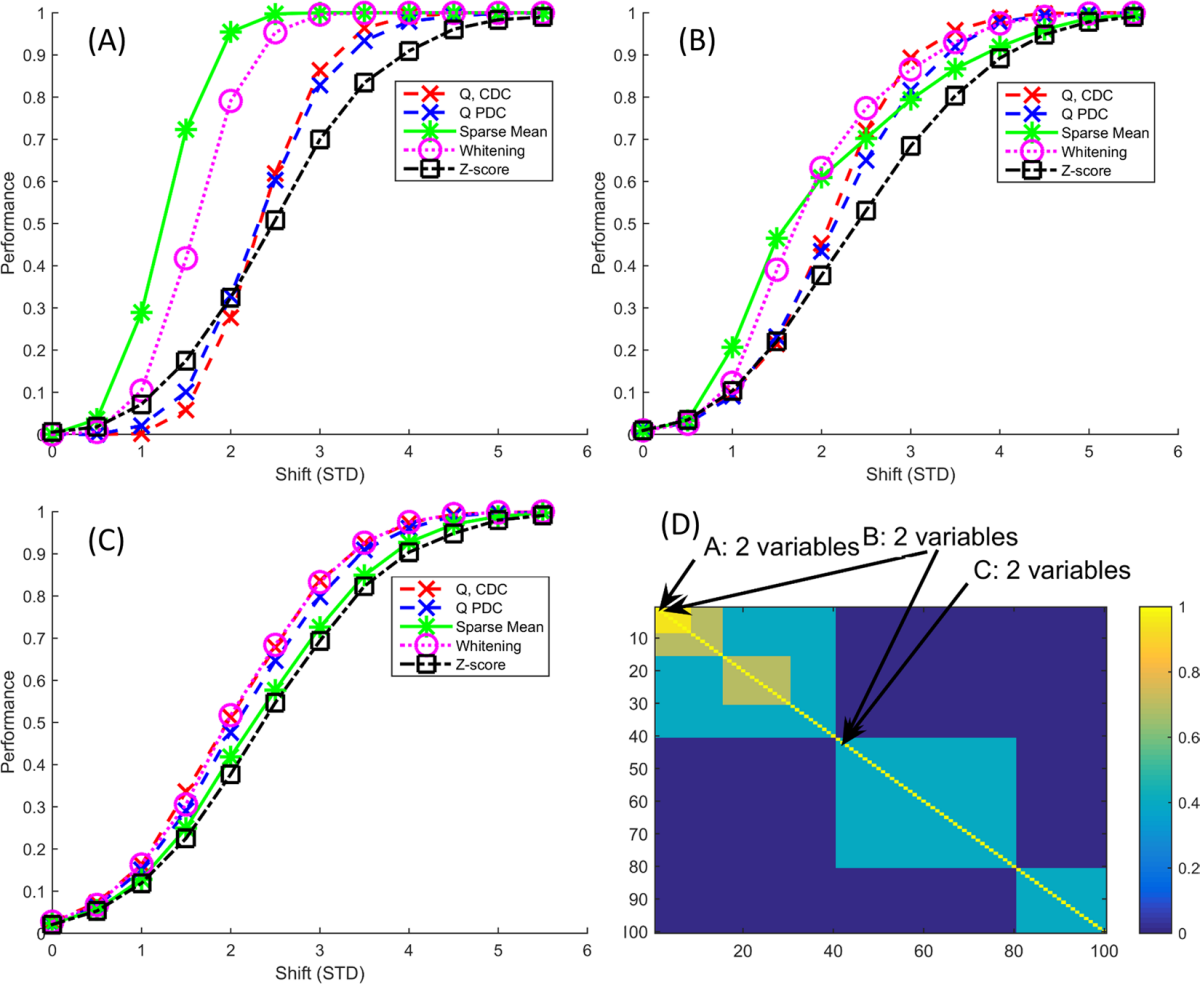
Results (**A**–**C**) from the more complex block correlation (**D**). (**A**) has the perturbation in the highly correlated first two variables while in (**B**) one of these same highly correlated values has been perturbed as well as one lowly correlated variable and (**C**) has the perturbation in two lowly correlated variables. These perturbations have been illustrated with arrows in (**D**). (**A**) is mostly analogous to the simple block structure (Fig. 1). (**B**) shows a different shape for the performance line of the Sparse Mean, this is due to the high diagnosis rate for the highly correlated variable together with relatively low diagnosis rate for the lowly correlated variable. See text for more details.

Next, we use a slightly more complex block covariance structure where the 2 abnormal variables are now in the highly correlated block (0.95) (Fig. 3A). Here we see much larger differences between the methods as the Sparse Mean, followed by the Whitening approach, perform much better than the other methods. For the small effect sizes, the *Z*-score outperforms the MSPC methods, although it performs still considerably poorer than Sparse Mean. The same correlation structure but with both perturbed variables now in the lowly correlated block (correlation 0.4) is shown in Fig. 3B. Here, the differences between the methods are subtler where the *Q*-contribution is better across all shift magnitudes but Whitening and the Sparse Mean are still competitive.

Comparison of these results to those where the perturbed variables were in the higher correlated block, shows that the ‘local’ correlation, the correlation of the perturbed variables to other variables, is a strong determining factor in the relative performance of the methods since the correlation structure remained the same in all three subfigures. This trend may be reproduced in all simulations: perturbed variables that are highly correlated to other variables may be recovered considerably better with the Sparse Mean or Whitening approach, while perturbed variables with low correlations to other variables may be marginally better recovered with the PCA-based MSPC methods. This indicates that, without prior knowledge on the actually perturbed variable and its relations to the other measured variables, the Sparse Mean or Whitening methods may be preferred. The strong correlation can assist in finding the abnormal variables as perturbing a variable that is strongly correlated with other variables ‘breaks’ this correlation, thus making it easier to identify. Strong correlations do not assist the *Q*-statistic in the same manner as it is assumed the residuals are uncorrelated (eq. 5).

This observation may be further confirmed by the situation where one ‘high correlation’ variable and one ‘low correlation’ variable has been made abnormal (Fig. 3C). The Sparse Mean returns the highly correlated variable with relative ease, while the uncorrelated variable is found in much fewer cases. For a shift of 2.5 the Sparse Mean finds the highly correlated variable every time and the other approximately half of the time, for the *Q*, no such difference exists. This is what causes the different shape in the performance of the Sparse Mean when compared with the other methods.

### Correlation structure based on real LC-MS metabolomics data

**Figure 4:**
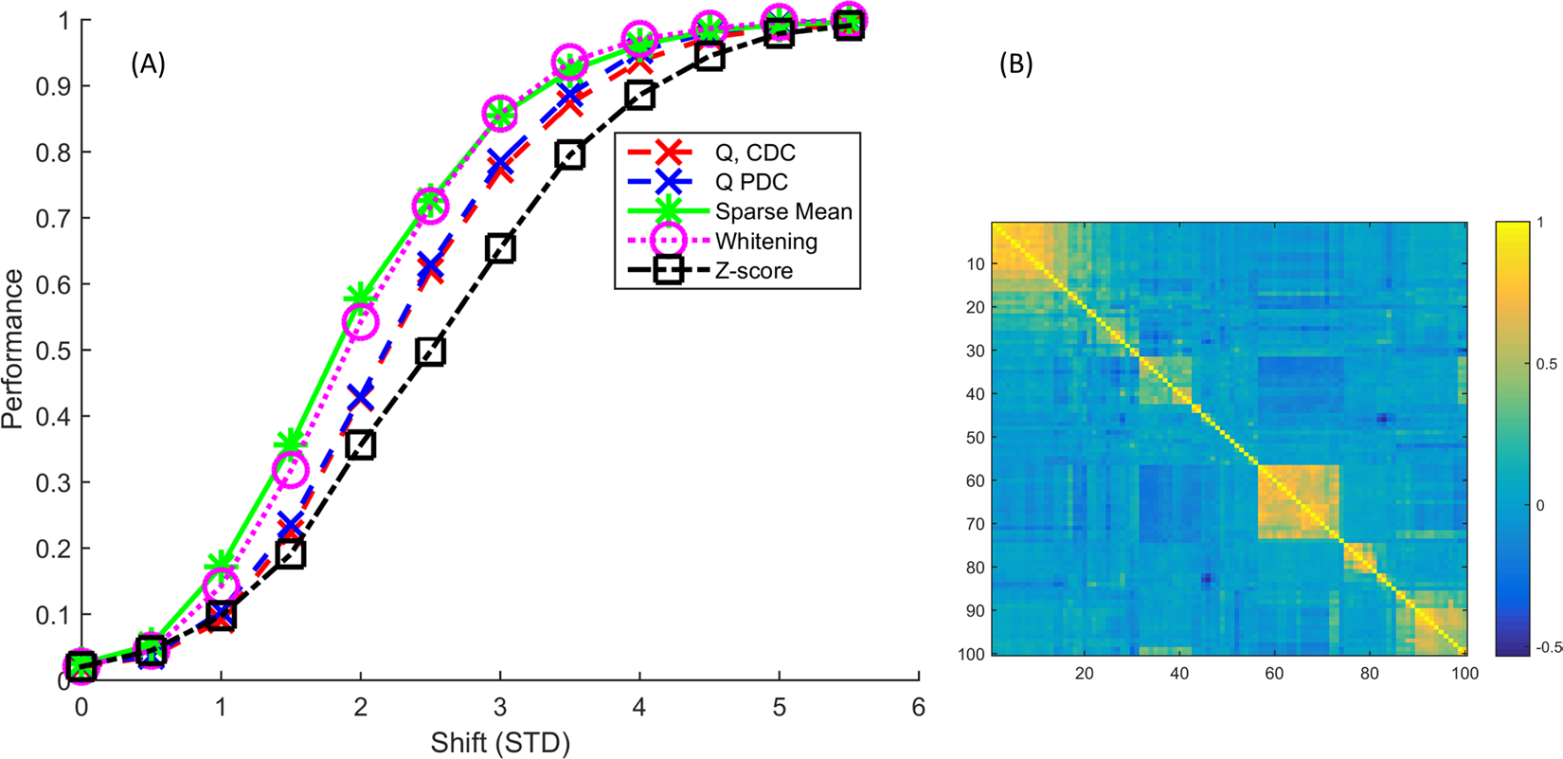
Performances for the HUSERMET project correlation structure (**A**) and the clustered correlation structure (**B**). Results are similar to the simple block correlation (Fig. 2).

Figure 4 shows the same methods on data from the real correlation structure from the HUSERMET project. We see the same pattern emerging as before: the Sparse Mean and Whitening are outperforming the MSPC methods, which in turn are outperforming the *Z*-score. It further shows our block correlation simulations are representative for a real correlation structure as Fig. 4 is quite similar to Fig. 2. We performed an extended version of this correlation structure but with 1000 variables instead of 100. While all used methods had decreased performances, their relative performances were very similar (results in Supplementary Materials).

### Metabolic network

**Figure 5:**
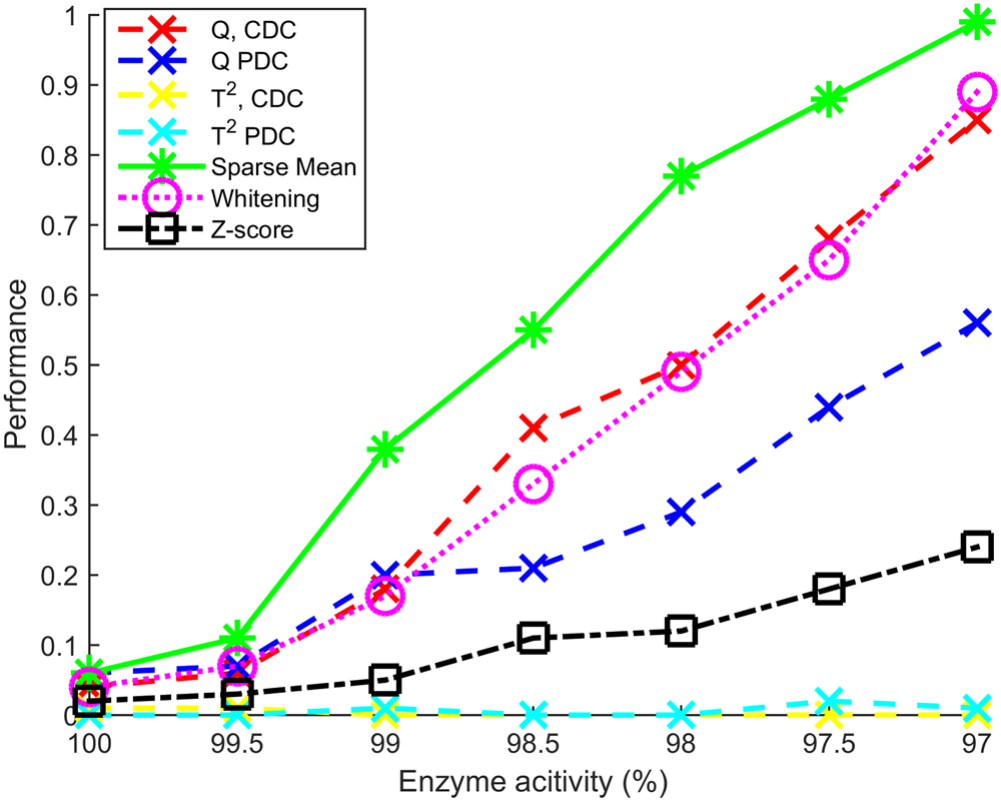
Performances for the metabolic network simulation for perturbed pyruvate decarboxylase. The enzyme rate constant has been decreased in steps where an enzyme activity of 100 represents the unperturbed network. The performances show the same pattern as with the block correlation structure where the Sparse Mean approach outperforms the others but the differences in performances is more pronounced due to the high correlation between variables in the network data.

Figure 5 shows the results of the metabolic network simulation for a perturbation in the pyruvate decarboxylase activity where the abnormal variable was pyruvate according to the Metabolic Control Analysis. We see the same pattern as with the block correlation structure where the Sparse Mean approach does best, followed by Whitening and the *Q*- statistic. The differences between the methods are however very large which may be caused by the high correlation (>0.99) between some of the variables. Notably, even with a relatively small perturbation such as 98% enzyme activity the Sparse Mean approach finds pyruvate as the most abnormal variable almost 100% of the time. This shows that Health Monitoring^7^ is promising as even small changes can be detected reliably even though its diagnosis is untargeted i.e. no prior information on classes is used. Strikingly, even though only one variable is abnormal, the univariate *Z*-score approach was outperformed by most multivariate approaches. This is due to the strong correlations: the multivariate models may detect a variable with a high value that is normal in a univariate sense as abnormal because variables that are highly correlated with it have a low value thus using correlations to detect it as abnormal.

### Fault diagnosis on real data

**Figure 6:**
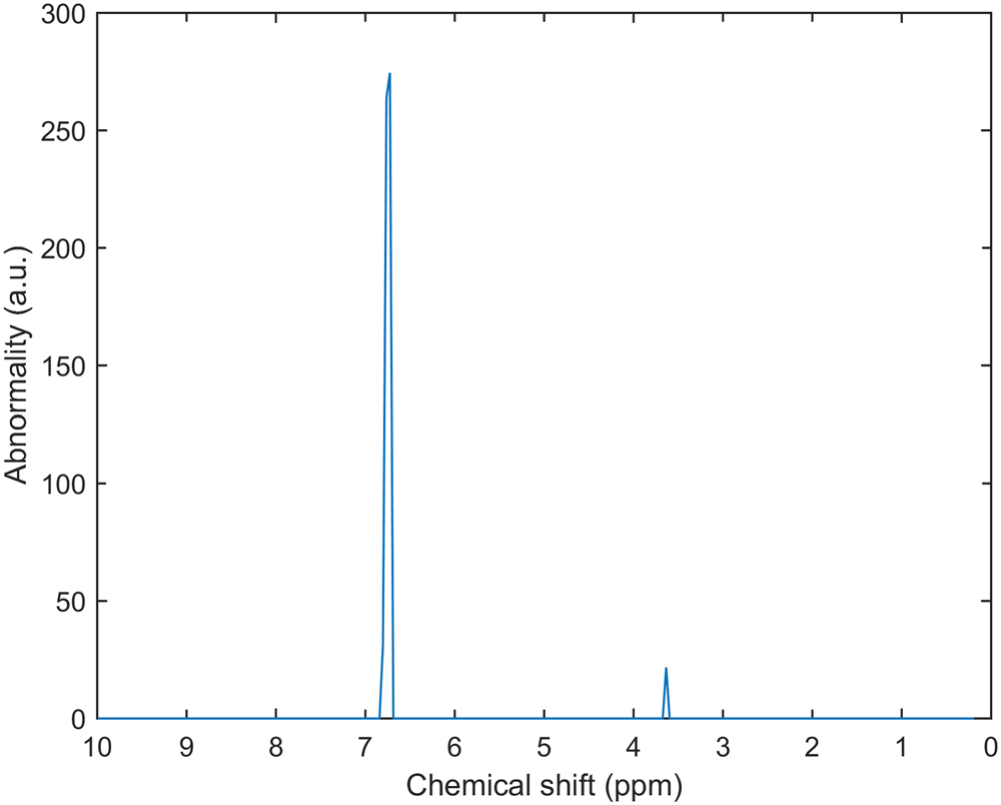
Example of the Sparse Mean approach for the diagnosis of alkaptonuria, a rare metabolic disease. The abnormality on the y axis corresponds to **u**_a_ from eq. (13). The two peaks identified correspond to Homogentisic acid, the metabolite which is known to occur high concentrations in urine in people suffering from alkaptonuria.

Figure 6 shows an example of the Sparse Mean applied on 1H-NMR urine data of children. Here we show an example of alkaptonuria, a rare metabolic disease is caused by a mutation which causes the body to accumulate homogentisic acid^29^ which in turn leads to several symptoms that may interfere with daily activities. The figure shows how the peaks associated with homogentisic acid are detected. This enables diagnosis of the disease. This example highlights the ease of interpretability due to the sparsity constraint on real data as well as how Health Monitoring may be applied for rare diseases for which limited examples are available.

## Discussion

The quality criterion we are using here only looks to the *l* most abnormal variables (2 in our case) and disregards the order of the rest. In our simulations we also employed other quality parameters, such as the mean rank of the ‘true’ abnormal variables in those identified by the methods, which led to very similar results. More simulations were run with different parameter settings, e.g. number of variables, blocksize, number of abnormal variables, etc. While the absolute performance decreases with, for example, increasing numbers of variables or decreasing the numbers of training samples, the relative performances of all methods remained similar such that the reported observations may be extended beyond the specifically selected simulation settings. This can partly be explained by the automatic procedure for optimizing *λ*, the shrinkage parameter. In situations with very few samples or many variables it is chosen relatively high. In the limit scenario where *λ* is chosen equal to 1, all methods perform equal as in Fig. 1. The Sparse Mean and Whitening approach may therefore always at least perform on par with the *Z*-score, at least in the simulation settings we attempted, but considerably outperform it in many scenarios within the evaluated simulation range. Therefore, we found no concrete limit to the number of variables for data to which these approaches may be applied. We would like to point out though, that detection of abnormal variables will still decrease with an increasing number of variables, making any approach potentially unusable.

In this work we only considered shrinkage towards the identity matrix. It may be agued^30^, that this may not be optimal and a different type of shrinkage could be considered instead i.e. imposing a sparse structure^31^ or non-linear shrinkage^30^. The covariance matrices obtained by such methods can be directly used in both the Sparse Mean and Whitening approach and may possibly even for PCA. This may improve detection of both abnormal samples and variables. A comprehensive overview of different covariance estimators can be found in Engel *et al*.^32^.

The methods discussed here use the assumption that the samples under investigation have a shift in their mean when compared to the control samples. This is not necessarily the only assumption possible. The method Sparse Statistical Health Monitoring for example uses the assumption that ${{\boldsymbol{\Sigma }}}^{-1}{({\bf{x}}-{\boldsymbol{\mu }})}^{{\bf{T}}}$ is sparse instead, this is like the assumptions made in several sparse Linear Discriminant Analysis methods^33^. While there are certainly merits to other assumptions we believe that the mean shift assumption is the most useful as allows for interpretation in the original variable space which is more straightforward. Furthermore, we found that we are better able to reproduce the results of the Metabolic Control Analysis using this assumption. Also, for analysis of the 1H-NMR data, it appeared that the method was quite sensitive to noise in the data (e.g. variables that define the baseline of the spectrum). The assumption that the data is normality distributed might not always (completely) hold for real data but it is still useful considering the often limited healthy data.

Overall, the regularized Sparse Mean approach seems to do very well in all scenario’s we investigated here. The *Q*-statistic and *T*^2^-statistic may be a reasonable choice as well, especially if there is an a priori reason to believe that the abnormality will manifest solely in the residual or model space. A univariate approach in the form of a *Z*-score often performs worse than the multivariate methods. The Whitening approach was almost always competitive with the best performing methods in our simulations, independent of the correlation structure used or which variables were perturbed. In addition to this, it allows for hypothesis testing which could be a huge benefit for e.g. biomarker discovery. In short, we recommend the Sparse Mean approach for most problems and Whitening if significance testing is required.

## Conclusion

Fault diagnosis is an important step after abnormality detection and has many applications. Here we have investigated different diagnosis methods based of the Mahalanobis distance such as the PCA-based Multivariate Statistical Process Control and three procedures based on variable selection. We found that the Whitening and Sparse Mean approach, which we combined with a shrinkage estimator of the covariance matrix have the best performance in our simulations. These combinations have not been published before to our knowledge.

The most important factor in the performance of these methods is how the abnormal variables are correlated to other normal and abnormal variables. We found that abnormal variable that have high correlation to other variables is best found using the Sparse Mean or Whitening methodology. In the absence of highly correlated variables the PCA-based methods have a slight edge. Assuming zero correlations in the form of calculating *Z*-scores is never better than the alternatives, even if the correlations are in fact zero. These observations hold significant promise for fault diagnosis in general and Health Monitoring more specifically which may be a step forward for disease detection.

## Supplementary information

Supplementary materials

## Data Availability

The Matlab scripts to generate the data for this study are available from the corresponding author on request.
