# Supplementary material for: Critical comparison of methods for fault diagnosis in metabolomics data
Source: Sci Rep. 2019 Feb 4;9:1123. doi: 10.1038/s41598-018-37494-7 (PMC6362212; doi:10.1038/s41598-018-37494-7)
Supplement: Supplementary file 1 — Supplementary materials [file 41598_2018_37494_MOESM1_ESM.docx]

**Critical comparison of methods for fault diagnosis in metabolomics data**

m.Koeman^1^, J. Engel^1,2^, J. Jansen^1^ and L. Buydens^1^

^1^Radboud University, Institute for Molecules and Materials (IMM)
Heyendaalseweg 135, 6525 AJ Nijmegen, The Netherlands
Email: [chemometrics@science.ru.nl](mailto:chemometrics@science.ru.nl)

^2^Biometris, Wageningen UR, Droevendaalsesteeg 1, 6708 PB Wageningen, The Netherlands

Supplementary materials

Derivation of $Z$-score from Mahalanobis distance

We start from the Mahalanobis distance:

$D_{m}^{2}(\mathbf{x})=\text{(}\mathbf{x}-\boldsymbol{\mu}\text{)}\boldsymbol{\Sigma}^{-1}{(\mathbf{x}-\boldsymbol{\mu)}}^{T}$ . (1)

We assume that $\boldsymbol{\Sigma=D}$ with $\mathbf{D}$ being the diagonal matrix with variances $\sigma_{i}^{2}$and write out the matrix multiplication as a sum leading to:

$D_{m}^{2}(\mathbf{x)})=\sum_{i=1}^{m} \text{(}x_{\boldsymbol{i}}-\mu_{i}\text{)}\frac{1}{\sigma_{i}^{2}} \text{(}x_{\boldsymbol{i}}-\mu_{i}\text{)}$ (2)

This can be simplified into the $Z$-score leading to:

$D_{m}^{2}=\sum_{i=1}^{M} Z_{i}^{2}$ with (3)
$Z_{i}=|\frac{x_{i}-\mu_{i}}{\sigma_{i}}|$ . (4)

1000 variable HUSERMET project correlation structure


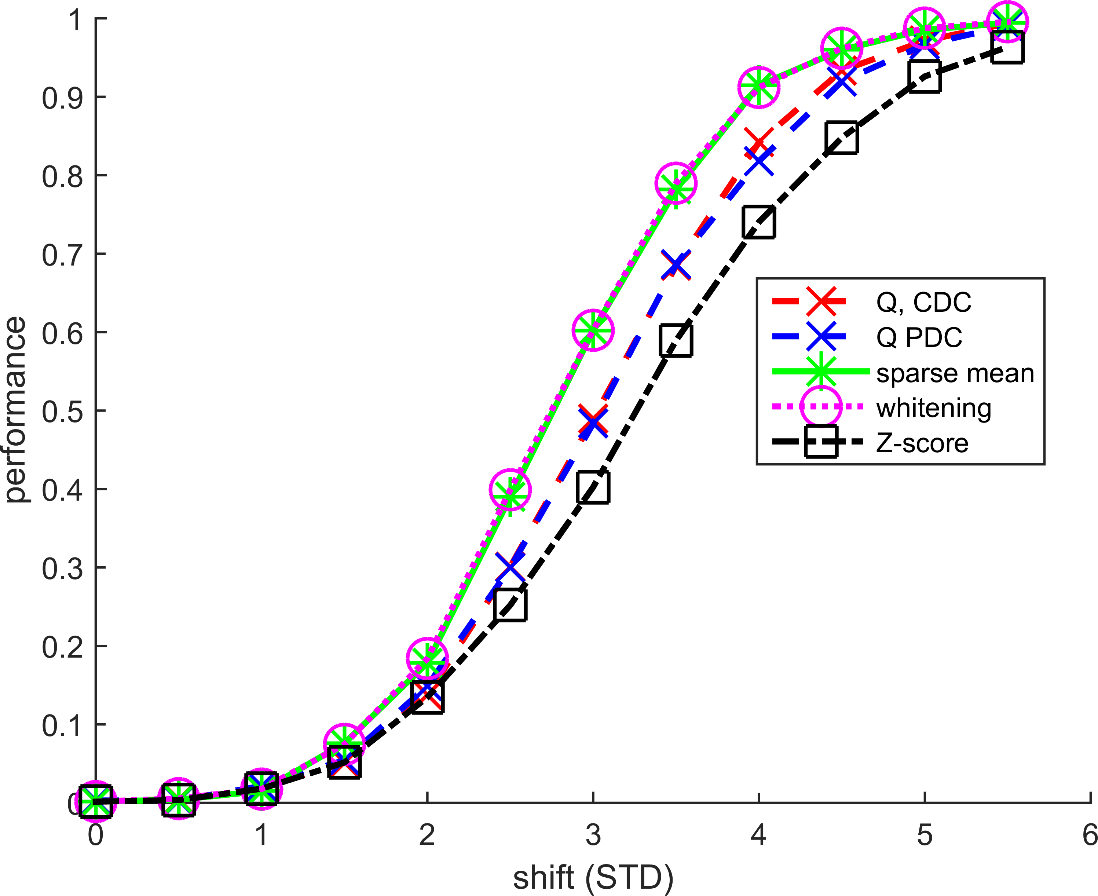


Supplementary figure 1: performances for the HUSERMET project correlation structure with 1000 variables. Even though the amount of variables has increased tenfold, the relative performances are very similar to those of figure 4.


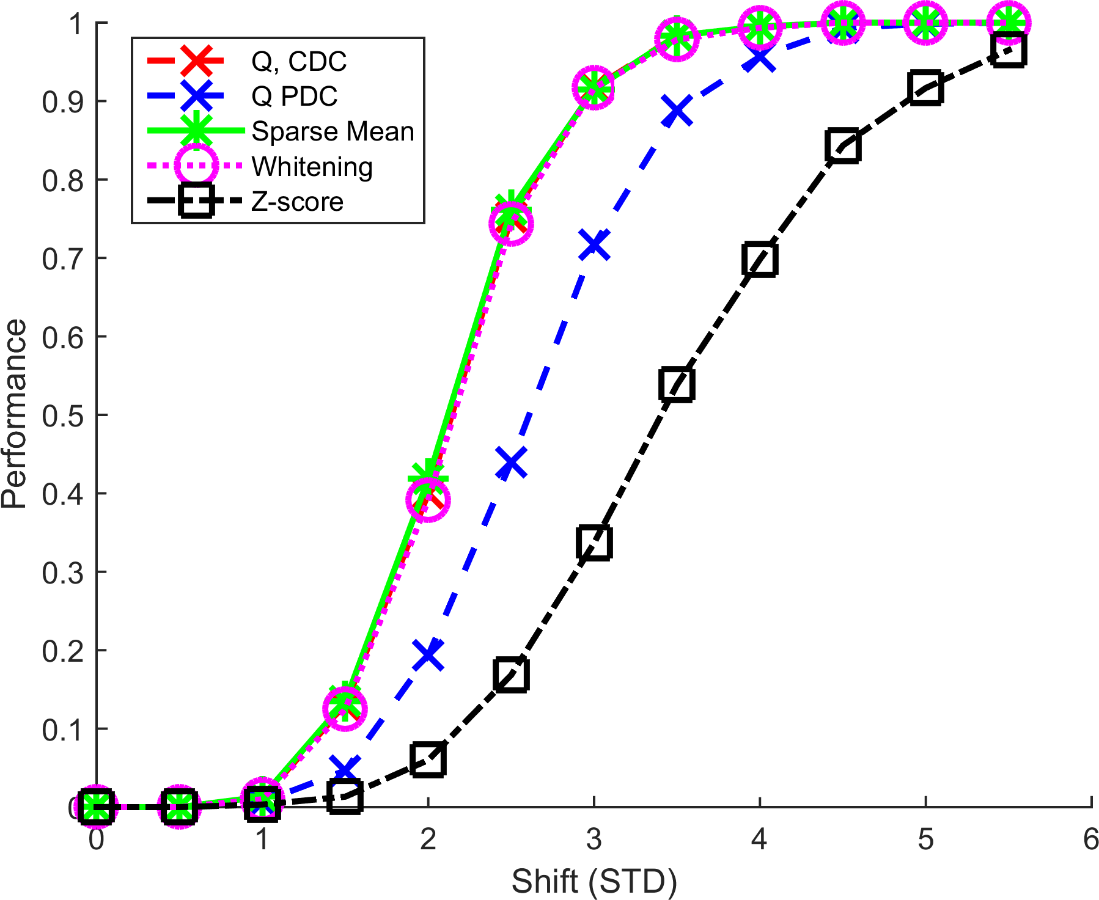


Supplementary figure 2: performances for the block simulation with 10 blocks of 200 variables with a correlation of 0.8 within the blocks. While the overall pattern is similar to that of Fig 2 in the results section, the three highest performing methods are more similar in performance.
